# Supplementary material for: Validation and refinement of existing methods for timed mating and early pregnancy detection in guinea pigs
Source: PLoS One. 2026 Apr 21;21(4):e0347318. doi: 10.1371/journal.pone.0347318 (PMC13098946; doi:10.1371/journal.pone.0347318)
Supplement: S1 File — (PDF) [file pone.0347318.s001.pdf]

Aug 19, 2025

Efficient timed mating and early pregnancy detection in guinea pigs

DOI
dx.doi.org/10.17504/protocols.io.kqdg31xpql25/v1

Marina Mayer[1], Ilja Finkelberg[2], Elvira Mass[1]

[1]Developmental Biology of the Immune System, Life and Medical Sciences (LIMES) Institute, University of Bonn, Bonn, Germany.;
[2]Children's Hospital, Pediatrics II, Pediatric Nephrology, University of Duisburg-Essen, Essen, Germany.

Marina Mayer
LIMES Institute

OPEN ACCESS

DOI: dx.doi.org/10.17504/protocols.io.kqdg31xpql25/v1

Protocol Citation: Marina Mayer, Ilja Finkelberg, Elvira Mass 2025. Efficient timed mating and early pregnancy detection in guinea pigs. **protocols.io** https://dx.doi.org/10.17504/protocols.io.kqdg31xpql25/v1

License: This is an open access protocol distributed under the terms of the **Creative Commons Attribution License**, which permits unrestricted use, distribution, and reproduction in any medium, provided the original author and source are credited

Protocol status: Working
We use this protocol and it's working

Created: August 15, 2025

Last Modified: August 19, 2025

Protocol Integer ID: 224744

Keywords: Timed mating, Guinea pigs, Estrous cycle, Vaginal cytology, Vaginal membrane, Pregnancy detection, Ultrasound, early pregnancy detection in guinea pigs guinea pig, structural similarity of the guinea pig placenta, guinea pig placenta, cytology examination for overnight mating, pregnancy detection, early pregnancy detection, overnight mating, guinea pigs guinea pig, embryonic developmental day, human placenta, mating, using vaginal impedance, mating efficiency, vaginal impedance, valuable animal models for various research question, valuable animal model, mice, using ultrasound

Funders Acknowledgements:
DFG
Grant ID: EXC2151-390873048
European Research Council (ERC)
Grant ID: 851257

Abstract

Guinea pigs have served as valuable animal models for various research questions. The structural similarity of the guinea pig placenta to the human placenta, particularly its hemomonochorial organization, supports its use in reproductive and developmental research. Yet, timed mating and early pregnancy detection remain significantly more challenging compared to mice, and many procedures are not standardized. Here, we present a comprehensive protocol to improve mating efficiency using vaginal impedance and cytology examination for overnight matings, and to enable pregnancy detection using ultrasound as early as embryonic developmental day (E) 12.

Attachments

DOCX

Protocol_Efficient t...

40KB

Image Attribution

<<Fig.1_Examination of the vaginal membrane.jpg>>, <<Fig.2_Vaginal impedence.jpg>>, <<Fig.3_Vaginal cytology.jpg>>, <<Fig.4_Mating time.jpg>>, <<Fig.5_Genotyping vaginal smear.jpg>>, <<Fig.6_Ultrasound examination.jpg>>, <<Fig.7_Weight changes.jpg>>

Materials

A	B	C
**Material**	**Article Number**	**Vendor**
**Animal equipment**		
Female Hartley Guinea pig	051	Charles River
Male Hartley Guinea pig	051	Charles River
Luftstromschrank UniProtect	5144450	Zoonlab
Irradiated meadow hay	WHEU	Altromin
Breeding diet for guinea pigs	3013 - 4mm pellets	Altromin
Vitamin C	02417	WDT Marktplatz
Cages	3045321	Zoonlab
**Vaginal cytology**		
FLOQSwabs	501CS01	Copan
DPBS, no calcium, no magnesium	11580476	Fisher Scientific
Microscope Slides with Ground Edges	11572203	Fisher Scientific
Methanol	1060351000	Merck
Pipette	Z331759-1PAK	Merck
Buffer tablets pH 7.2 for preparing buffer solution acc. to WEISE	109468	Merck
May-Grünwald’s eosine-methylene blue solution modified	1014240100	Merck
Giemsa’s azur eosin methylene blue solution	1092040100	Merck
DPX Mountant for histology	06522-100ML	Merck
Cover glasses	BRND470819	Avantor
**Ultrasound examination**		
Long hair trimmer	2997703	Conrad Electronic SE
Ultrasound gel	140603	Intermed
Butterfly iQ+ Vet		Butterfly Network
Vscan Air™ CL		GE Healthcare

A	B	C
**PCR**		
DreamTaq™ Green PCR Master Mix (2X)	K1082	Thermo Fisher Scientific
OneTaq® DNA Polymerase	M0480L	NEB
DEPC Water DNase/RNase-Free Distilled Water	10977035	Thermo Fisher Scientific
Primer Working Stocks<br><br>solve with DEPC Nuclease free water	See sequence in publication (1)	Microsynth
PCR tubes	211-3263	VWR
PCR tube lids	731-1250	VWR
UltraPure™<br>TBE-buffer (10X)<br><br>TBE buffer (10x) powder	15581028<br><br><br>A4348,9010	Thermo Fisher Scientific<br><br>Applichem
Ultrapure Agarose	10264544	Fisher Scientific
Sybr Safe/<br>peqGREEN 20 000x DNA/RNA-dye, 2×1 ml	732-3196	VWR
100 bp DNA Ladder<br>Mix 1+5 with violett dye (diluted)	N3231S	New England Biolabs (NEB)
DirectPCR-Lysis Reagent (Mouse Tail)	102-T	Qiagen
Proteinase K-solution (20 mg/mL)	4392-0005	PanReac AppliChem
All Prep DNA/RNA Micro Kit (50)	80284	Qiagen
**General Equipment**		
Chemical fume hood		
Lightmicroscope		
Animal scale		
Coplin Jars		
4°C refrigerator		
pH Indicator		
Thermal cycler		
Agarose Gel system		
Gel Imager		
NanoDrop		
Double-distilled water		

Buffers

WEISE buffer
1 Buffer tablet pH 7.2 in 1 l double distilled water
→ measure pH and adjust accordingly
May-Grünwald working solution (Volume for 100 ml)
May-Grünwald's eosine-methylene blue solution modified: 15 ml
WEISE buffer solution: 10 ml
Deionised water: 75 ml
→ Let the solution stand for 10 minutes, then filter before use.
Giemsa working solution (Volume for 100 ml)
Giemsa's azur eosin methylene blue solution: 5 ml
WEISE buffer solution: 95 ml
→ Let the solution stand for 10 minutes, then filter before use.

Safety warnings

This protocol contains hazardous chemicals including methanol, methylene blue, Eosin Y and Azure B. Appropriate PPE and institutional-specific safety precautions including appropriate waste disposable should be taken.

Ethics statement

All investigations concerning animal work have local approval and all procedures conform to the guidelines from Directive 2010/63/EU of the European Parliament on protecting animals used for scientific purposes. Animal procedures were performed in adherence to the project license issued by the Landesamt für Natur, Umwelt und Verbraucherschutz (LANUV, Az.nr: 81-02.04.2020.A310).

Vaginal membrane monitoring

1 Acquire Hartley guinea pigs no younger than 2 months of age. Female guinea pigs typically reach sexual maturity at 2–3 months, and the first pregnancy should ideally occur before 6–7 months of age (2).

2 Allow guinea pigs to acclimate for at least two weeks in your facility before initiating regular handling. Ensure all relevant Animal Care and Welfare guidelines are followed, including a 12-hour light/dark cycle, ambient temperatures of 21 ± 2 °C, and relative humidity of 55% ± 15% (3). Guinea pigs must be checked and fed daily, with food and water provided *ad libitum*.

Note

Guinea pigs have relatively weak intestinal peristalsis, making constant access to hay essential for maintaining gastrointestinal motility (4). Additionally, guinea pigs cannot synthesize vitamin C and require dietary supplementation (5).

Note

Separate male guinea pigs 2–3 days prior to mating to reduce stress and aggressive behavior. This may also help with reuniting the males later.

3 Record daily observations of the vulva and vaginal membrane, noting color, swelling, and membrane integrity. This examination can be performed by one or two people. To restrain the guinea pig, support the guinea pig with one hand beneath the forelimbs and the other under the pelvic region. Use the little finger of the upper hand to gently lift the genital region upward, and the thumb and index finger of the lower hand to carefully part the labia (Fig. 1).

4

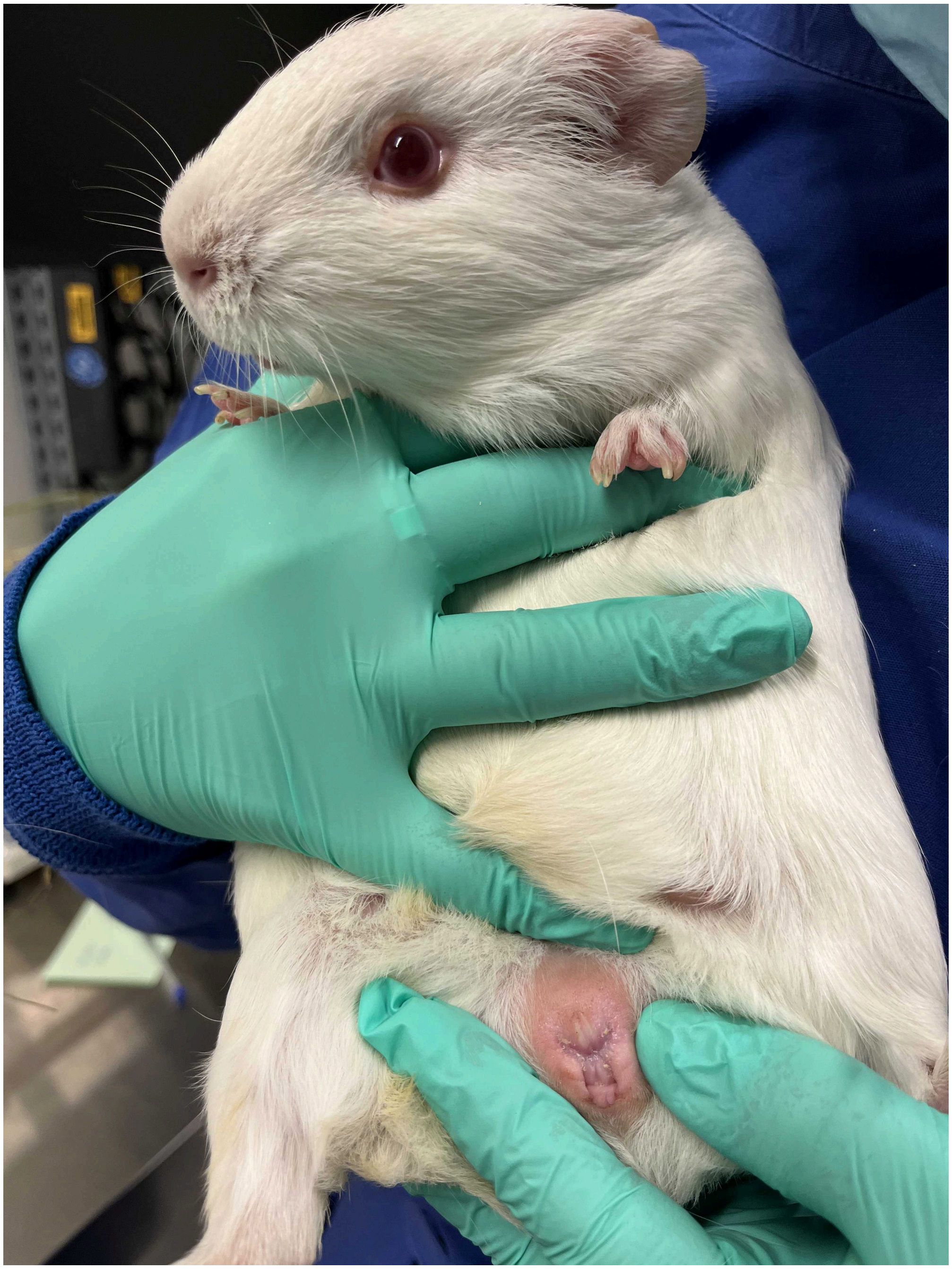

Figure 1. Examination of the vaginal membrane.

5 The vaginal membrane is considered **closed** when it appears visibly pale (Fig. 2A). This phase is the longest in the estrous cycle and typically lasts around 9 days (Fig. 2B). During the **opening** phase, which usually lasts only one day, the vulva begins to swell and the membrane changes colour from pale to light pink (Fig. 2A). Once the vaginal membrane is fully **open**, it appears ruptured, with a dark pink coloration and pronounced vulvar swelling (Fig. 2A). After approximately 4 days, the membrane begins to **close**, starting from the edges and leaving a small pink opening visible (Fig. 2A). Over the next

~3 days, it fully seals again. The full estrous cycle in guinea pigs lasts approximately 15–17 days (Fig. 2B).

6 Since the estrous only lasts for approximately 6–11 hours (6), optimal timing for mating is the first day of membrane opening, particularly when the membrane was observed as closed the previous day.

Note

The initial “opening” stage may be difficult to identify precisely. Therefore, daily documentation of vulvar changes is essential. Mating should be attempted at the first visible signs of opening, even if the membrane already appears ruptured.

Note

A persistently closed vaginal membrane over several weeks may indicate successful mating and pregnancy, but its reliability for pregnancy confirmation is limited due to occasional spontaneous perforation during gestation.

7

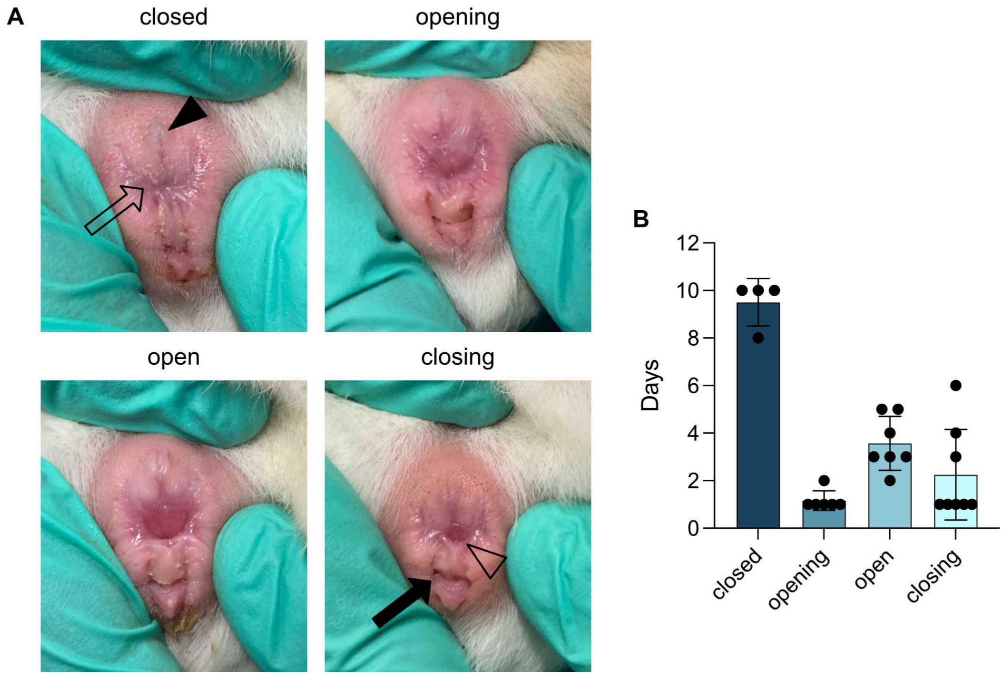

Figure 2. Changes in the vaginal membrane during different phases of the estrous cycle. (A) Closed vaginal membrane: The female external genitalia form a visible trident shape when the vaginal membrane is closed. Opening: The vaginal membrane begins to separate, and the clitoris appears swollen and changes colour from pale to a darker pink. Open: The vaginal membrane is fully perforated and the external vaginal opening is visible as a dark pink opening. Closing: The vaginal membrane begins to close from the outer edges, and the swelling subsides. Filled arrowhead: urethral opening, open arrow: vaginal membrane, filled arrow: anus, open arrowhead: vaginal opening. (B) Duration of each phase of the estrous cycle based on changes in the vaginal membrane (each dot represents one animal; mean ± SD).

Vaginal cytology

8 To further increase mating efficiency, collect a vaginal smear on the day the female is to be paired for mating. Moisten a sterile swab with sterile phosphate-buffered saline (PBS) or 0.9% saline and gently insert it into the vulva. Avoid deep insertion to prevent trauma. Rotate the swab gently three times and transfer the collected cells onto a clean glass slide and let it dry for 10 minutes.

> **Note**
>
> Vulvar spreading is not required for this procedure. Always inspect the vaginal membrane beforehand, as deep swab insertion during membrane closure can cause rupture.

9 Fix the cells by applying 2–3 drops of methanol directly to the slide. Allow the methanol to evaporate completely under a chemical fume hood. Slides can be stained immediately or stored at 4 °C for several weeks.

10 Perform May-Grünwald-Giemsa staining:

1. Place the fixed slides in a Coplin jar containing May-Grünwald working solution (prepared with 15% May-Grünwald solution, 10% WEISE buffer solution (pH 7.2) in 75% deionised water) for 6 minutes.
2. Rinse the slides twice in WEISE buffer solution (pH 7.2), 1 minute each.
3. Transfer the slides into Giemsa working solution (prepared by diluting 5% Giemsa solution in WEISE buffer solution (pH 7.2)) for 20 minutes.
4. Rinse again twice in WEISE buffer solution (pH 7.2), 1 minute each.
5. Assess staining under a light microscope. If staining appears too weak, repeat the staining procedure.
6. If satisfactory, mount the slide with a coverslip using a non-aqueous mounting medium (e.g., DPX) and let it dry.

> **Note**
>
> Other cytological stains, such as Papanicolaou, may also be suitable for identifying the major vaginal epithelial cell types.

11 Observe the stained slide under a light microscope. Five distinct vaginal cell types can typically be identified in guinea pig smears (Fig. 3A):

1. Leukocytes: Small, polymorphonuclear cells with nearly colourless cytoplasm.
2. Parabasal cells: Round cells with a clearly defined membrane and a large, centrally located nucleus.
3. Intermediate cells: Cells with a smaller nucleus and irregular cell borders.
4. Superficial cells: Cells with faintly stained cytoplasm and fading nuclei.

5. Anuclear cells: Large, flat cells lacking visible nuclei and with poorly defined membranes.

Note

The distribution of these cells correlates with the estrous cycle. Leukocytes and parabasal cells are most abundant during the opening phase, decrease through the open and closing phases, and are largely absent during the second half of the closed phase of the cycle (Fig. 3B).

12

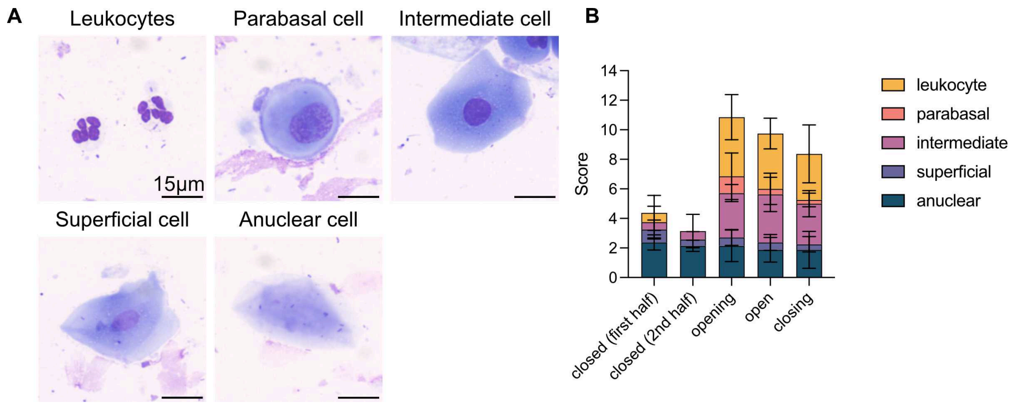

Figure 3. Vaginal cytology across the estrous cycle. (A) Five distinct cell types can be identified in a vaginal smear. Leukocytes are small, polymorphonuclear cells with a nearly colourless cytoplasm. Parabasal cells are round with a clearly defined cell membrane and a large nucleus. Intermediate cells have a smaller nucleus and an irregular cell membrane. Superficial cells show fading cytoplasm and a diminishing nucleus. Anuclear cells lack a visible nucleus, and their cell membranes are often poorly defined. (B) The distribution of these cell types varies over the course of the estrous cycle (n = 7-8).

13 Smears are often unevenly distributed; therefore, scan the entire slide to identify all present cell types. If necessary, capture five representative images to assist with interpretation and documentation. Score the smear using the scoring system shown in Figure 4A, and refer to the scheme in Figure 4B to assess the likelihood of pregnancy. The presence of leukocytes and parabasal cells usually indicates that the animal has already progressed through estrous (6). In contrast, a high score of intermediate and/or superficial cells can be indicative of a higher likelihood of pregnancy (Fig. 4D). Mucus appears as purple-stained streaks with the May-Grünwald-Giemsa staining and serves as a useful indicator of potential fertility (Fig. 4C). Mating is typically carried out for 12–24 hours, often overnight, to align with peak estrous (6).

Note

Anuclear cells are commonly present in most smears. However, anuclear, superficial, and intermediate cells become smaller and highly fragmented just before or during estrous. Nuclei without associated cytoplasm may also be observed (Fig. 4C, arrowhead).

14 One day after mating, guinea pigs that have conceived may continue to progress through the estrous cycle, and leukocytes and parabasal cells may appear. In contrast, guinea pigs with unsuccessful conception tend to lose parabasal cells and leukocytes from the smear (Fig. 4E).

Of note: Attempts were made to detect sperm or other male cells in vaginal smears to confirm coitus using PCR for X and Y chromosome sequences. Published primers (1) successfully amplified DNA from liver tissue (Fig. 5) and sperm (data not shown), however, the material obtained from vaginal smears was insufficient for reliable amplification, despite testing multiple purification and lysis protocols.

15

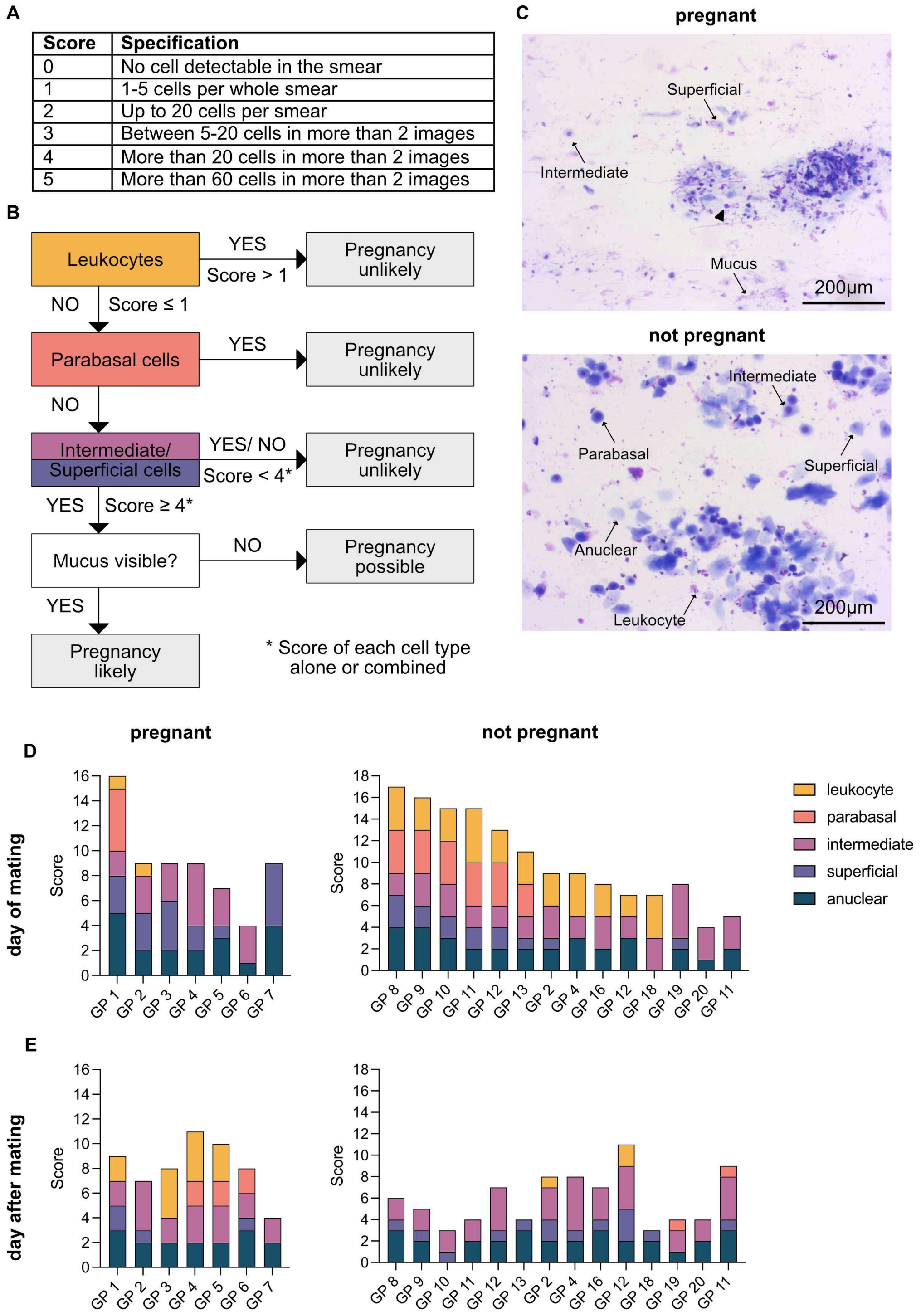

Figure 4. Vaginal cytology as a measure of fertility. (A) Scoring system based on the relative presence of different vaginal cell types. (B) Decision-making scheme to assess the likelihood of a positive pregnancy outcome. (C) Representative vaginal smears from the day of mating in one pregnant and one non-pregnant animal. In the pregnant animal, fragmented cells and mucus are visible, whereas in the non-pregnant animal, large, well-defined cells predominate. (D) Distribution of cell types on the day of mating in animals that were later confirmed to be pregnant or failed to conceive. (E) Changes in cell type distribution one day after mating in animals that were later confirmed to be pregnant or failed to conceive.

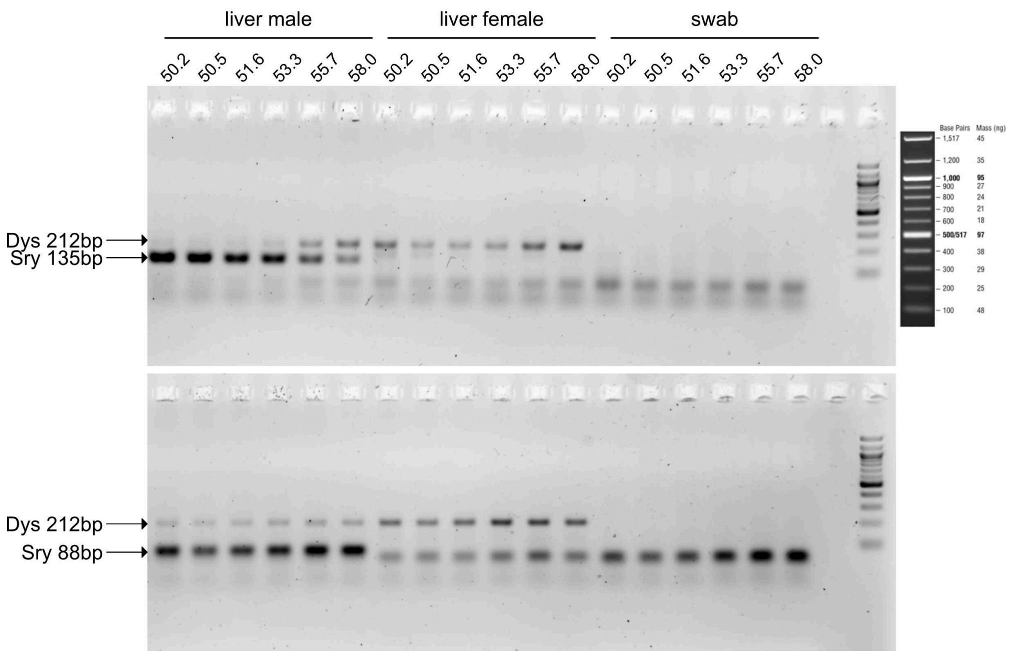

Figure 5. Insufficient DNA yield from vaginal smears for sex chromosome PCR detection. Agarose gel showing PCR amplification using a temperature gradient on male and female liver tissue samples. The expected 212 bp band corresponds to Dystrophin (X chromosome), and the 135 bp and 88 bp bands correspond to Sry (Y chromosome). Male liver tissue shows bands for both X and Y chromosomes, while female liver tissue shows only the X-chromosome band. No specific bands are detected in DNA extracted from the vaginal smear sample, indicating insufficient DNA for detection. Note that faint, non-specific bands, likely primer dimers, appear in most samples and migrate below 88 bp.

Ultrasound examination

16 Early pregnancy detection is possible via transabdominal ultrasound as early as E12. The procedure should be performed by two trained personnel experienced in handling the guinea pigs. Prepare a soft surface using towels for the guinea pig to lie on in an empty cage or on an examination table, and have a clean towel ready to remove excess ultrasound gel after the procedure.

17 Shaving the abdomen is required for optimal image quality. One person should hold the guinea pig upright while the other uses an electric razor to shave the area from the caudal ribs to the cranial pelvic region (see Fig. 6A, dashed outline). Take care to avoid injury to the mammary glands during shaving.

> **Note**
>
> Shave generously, as re-shaving is not feasible once gel has been applied. If the guinea pig is accustomed to human handling, sedation is not required for either shaving or ultrasound examination.

18 One person gently restrains the guinea pig securely in an upright lying position, holding it under the forelimbs. Covering the head with a towel can reduce visual stimuli and help relax the guinea pig. The second person performs the ultrasound from below, supporting the hind limbs with one hand and controlling the probe with the other.

19 Apply a generous amount of ultrasound gel to the shaved abdomen. Press the probe gently onto the skin and adjust depth and contrast settings to optimize visualization of internal structures. Figure 6B-C compares the imaging quality of two ultrasound devices. Embryos at E12 can be visualized using both the Butterfly iQ+ Vet (Butterfly Network, Inc.) and the VScan Air CL (GE HealthCare); however, the VScan Air CL provides superior contrast and sharper image quality.

20 Place the probe in a transverse (cross-sectional) orientation just cranial to the external genitalia. A full urinary bladder appears as a large, anechoic (black), round structure and is often the first identifiable organ. Continue scanning cranially. The uterus lies dorsal to the bladder and bifurcates laterally into two uterine horns. Gestational sacs typically appear as anechoic, spherical structures with a surrounding hyperechoic (white) rim. Continue scanning cranially along both uterine horns, as embryos may be located in one or both horns and can be positioned relatively cranially. At E12, the gestational sac measures approximately 1 cm in diameter, with the surrounding wall measuring 2–3 mm in thickness. Placental tissue (Fig. 6C, hollow arrow), and the embryo (Fig. 6B, arrowhead) may also be visible within the gestational sac as echogenic (white) structures. The upper extent of the uterus is reached when kidney or liver tissue become visible.

Note

If the probe is placed too cranially, the portal vein within the liver can sometimes be mistaken for an embryo due to its prominent, echogenic wall.

21 After the examination, gently remove the gel with a dry towel and return the guinea pig to its cage.

22

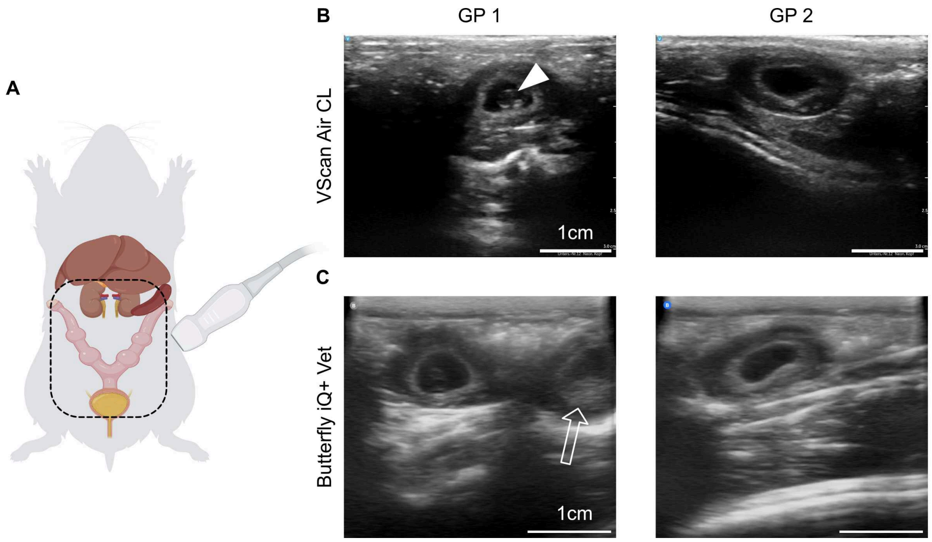

Figure 6. Ultrasound examination of embryos at E12. (A) Schematic showing the anatomical position of internal organs and the pregnant uterus. The dashed outline indicates the area that must be shaved prior to ultrasound examination. (B) Ultrasound images from two pregnant animals obtained using the VScan Air CL, the filled arrowhead indicates a visible embryos. (C) Ultrasound images from two pregnant animals obtained using the Butterfly iQ+ Vet, the open arrow indicates a visible placenta.

Pregnancy progression and embryonic development

23 Pregnancy in guinea pigs can only be reliably detected through weight gain after gestational week 6 (Fig. 7A). In non-pregnant females, average weight gain is approximately 1–2% per week, whereas in pregnant females, weight gain increases to 5–10% per week in the second half of pregnancy.

Note

These weight changes were not observed to be influenced by the number of offspring or the initial body weight of the females at the time of mating.

24 While guinea pig pregnancies may begin with 2–5 embryos, some embryos appear to undergo resorption after E27, as litter size at birth typically ranges from 1 to 4 pups (Fig. 7B).

25

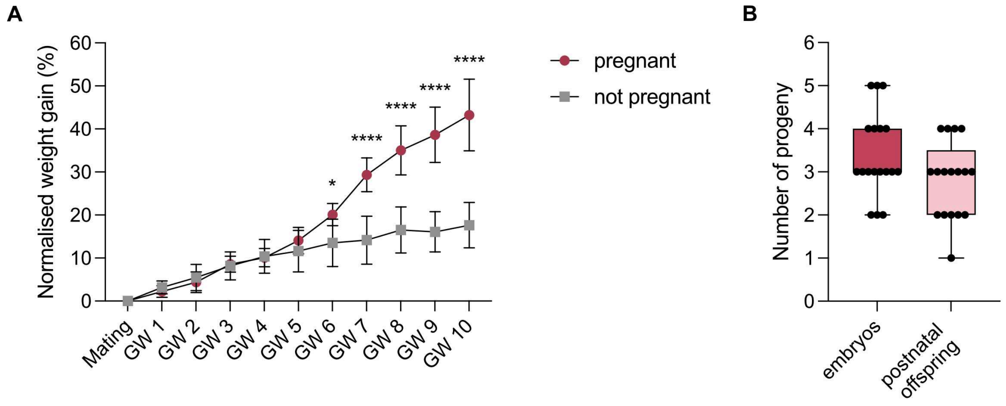

Figure 7. Weight gain in pregnant versus non-pregnant guinea pigs and litter size. (A) Normalized weight gain over time in pregnant (n = 7) and non-pregnant (n = 9) female guinea pigs. A significant increase in weight gain is observed in pregnant animals beginning at gestational week 6 (* $p < 0.05$), based on multiple unpaired t-tests with Welch's correction. (B) Comparison of embryo counts during gestation (days 11-27) and the number of live-born offspring per dam; each dot represents one animal. Error bars represent mean ± SD. Statistical significance: * $p < 0.05$, **** $p < 0.0001$.

Protocol references

1. Depreux FF, Czech L, Whitlon DS. Sex genotyping of archival fixed and immunolabeled guinea pig cochleas. Sci Rep. 2018 Mar 26;8(1):5156.

2. Genzer SC, Flietstra T, Coleman-McCray JD, Tansey C, Welch SR, Spengler JR. Effect of Parental Age, Parity, and Pairing Approach on Reproduction in Strain 13/N Guinea Pigs (Cavia porcellus). Animals (Basel). 2023 Mar 1;13(5).

3. Wilson RL, Lampe K, Matushewski BJ, Regnault TRH, Jones HN. Time Mating Guinea Pigs by Monitoring Changes to the Vaginal Membrane throughout the Estrus Cycle and with Ultrasound Confirmation. Methods Protoc. 2021 Aug 27;4(3).

4. Nicholas S, Spencer NJ. Peristalsis and fecal pellet propulsion do not require nicotinic, purinergic, 5-HT3, or NK3 receptors in isolated guinea pig distal colon. Am J Physiol Gastrointest Liver Physiol. 2010 Jun;298(6):G952-61.

5. National Research Council (US) Subcommittee on Laboratory Animal Nutrition. Nutrient Requirements of the Guinea Pig. 1995;

6. (PDF) The Guinea pig estrous cycle: Correlation of vaginal impedance measurements with vaginal cytologic findings [Internet]. [cited 2025 Jul 2]. Available from: https://www.researchgate.net/publication/13796172*The*Guinea*pig*estrous*cycle*Correlation*of*vaginal*impedance*measurements*with*vaginal*cytologic*findings#fullTextFileContent

Acknowledgements

We thank Cornelia Cygon, Dr. Nelli Blank-Stein, Dr. Maria Römelt, Dr. Linda Müller, Nikola Makdissi, Dr. Katharina Mauel, Eliana Franco Taveras and Theresa Eulgem for their support with guinea pig handling and Dr. med. Metin Cetiner for his help with ultrasound examination.
